# Mechanistic model for predicting the seasonal abundance of *Culicoides* biting midges and the impacts of insecticide control

**DOI:** 10.1186/s13071-017-2097-5

**Published:** 2017-03-27

**Authors:** Steven M. White, Christopher J. Sanders, Christopher R. Shortall, Bethan V. Purse

**Affiliations:** 1 Centre for Ecology & Hydrology, Benson Lane, Wallingford, Oxfordshire, OX10 8BB UK; 2 Wolfson Centre for Mathematical Biology, Mathematical Institute, Radcliffe Observatory Quarter, Woodstock Road, Oxford, Oxfordshire, OX2 6GG UK; 3 The Pirbright Institute, Ash Road, Pirbright, Surrey, GU24 0NF UK; 4 Rothamsted Research, Harpenden, Herts, AL5 2JQ UK

**Keywords:** Bluetongue, Deltamethrin, Obsoletus group, Schmallenberg, Seasonality, Vector-borne disease

## Abstract

**Background:**

Understanding seasonal patterns of abundance of insect vectors is important for optimisation of control strategies of vector-borne diseases. Environmental drivers such as temperature, humidity and photoperiod influence vector abundance, but it is not generally known how these drivers combine to affect seasonal population dynamics.

**Methods:**

In this paper, we derive and analyse a novel mechanistic stage-structured simulation model for *Culicoides* biting midges-the principle vectors of bluetongue and Schmallenberg viruses which cause mortality and morbidity in livestock and impact trade. We model variable life-history traits as functional forms that are dependent on environmental drivers, including air temperature, soil temperature and photoperiod. The model is fitted to Obsoletus group adult suction-trap data sampled daily at five locations throughout the UK for 2008.

**Results:**

The model predicts population dynamics that closely resemble UK field observations, including the characteristic biannual peaks of adult abundance. Using the model, we then investigate the effects of insecticide control, showing that control strategies focussing on the autumn peak of adult midge abundance have the highest impact in terms of population reduction in the autumn and averaged over the year. Conversely, control during the spring peak of adult abundance leads to adverse increases in adult abundance in the autumn peak.

**Conclusions:**

The mechanisms of the biannual peaks of adult abundance, which are important features of midge seasonality in northern Europe and are key determinants of the risk of establishment and spread of midge-borne diseases, have been hypothesised over for many years. Our model suggests that the peaks correspond to two generations per year (bivoltine) are largely determined by pre-adult development. Furthermore, control strategies should focus on reducing the autumn peak since the immature stages are released from density-dependence regulation. We conclude that more extensive modelling of *Culicoides* biting midge populations in different geographical contexts will help to optimise control strategies and predictions of disease outbreaks.

**Electronic supplementary material:**

The online version of this article (doi:10.1186/s13071-017-2097-5) contains supplementary material, which is available to authorized users.

## Background

Vector-borne diseases account for a large proportion, approximately 30%, of the World’s emerging infectious diseases, and the rate of emergence is on the increase [1]. Vector abundance and seasonal dynamics are important determinants of variation in the risk of vector-borne infections [2–4] and are both influenced by climate. As well as producing seasonal and spatial variation in the ratio of vectors to hosts, vector dynamics govern the time delay between the acquisition and transmission of a pathogen by adult vectors, since within-vector pathogen replication (extrinsic incubation period: EIP) is largely determined by temperature [5–7] and not all vectors will survive to bite again once this period has been completed [8]. In vector-borne pathogens where transmission is primarily horizontal (not between successive life-stages or generations of the vector), the likelihood that pathogens will persist between transmission seasons in temperate zones is dependent on whether adult vectors can survive, and maintain continual vector-to-host-to-vector transmission cycles, over winter [9]. For other midge-borne pathogens, disease impacts may depend on whether infection by adult vectors occurs during a critical window in the seasonal reproductive cycle of host animals (Schmallenberg virus infection must occur in a particular period of pregnancy in host ruminants to result in abortions or congenital malformations [10, 11]).

*Culicoides*-borne pathogens are emerging and increasing in incidence and impact worldwide, likely due to multiple environmental change drivers including climate, land use, trade and animal husbandry [12]. Transmitted (horizontally) by *Culicoides* biting midges (Diptera: Ceratopogonidae), their transmission patterns are highly seasonal, especially in temperate zones, and have been linked to adult vector dynamics [6, 13]. Disease control measures include vaccination of susceptible ruminant livestock and restrictions on the movement and trade of livestock (e.g. enshrined for the EU under Council Directive 2000/75/EC) and vector control [14]. Though these measures reduce the speed and extent of spread of viruses, they impose huge logistic and welfare costs on affected regions and so tend to be restricted seasonally according to the adult vector season. For example, vaccination and relaxation of affected livestock restrictions occur during winter, when nightly light-suction trap catches of *Culicoides* fall below a threshold (the ‘seasonally vector free period’: SVFP). Therefore, understanding seasonal dynamics of *Culicoides* is critical to understanding and predicting persistence and spread of these emerging, high impact livestock diseases, particularly in temperate zones, and to optimising mitigation strategies. This understanding is particularly critical for Europe which has suffered tens of thousands of outbreaks due to multiple incursions and rapid spread of bluetongue virus strains [15, 16] and the emergence and spread of a novel Orthabunyavirus, Schmallenberg virus [17].

Furthermore, the effectiveness of vector control as a mitigation strategy can only be improved by elucidating the mechanisms underpinning midge population dynamics. Insecticides applied to host animals have shown some potential for causing high mortality in host-seeking females. A recent study determined the susceptibility of European *Culicoides* species to deltamethrin in a field trial consisting of a pour-on formulation of the insecticide applied to sheep [18]. They found a maximum mortality rate of 49% at 4 days post-application, and duration of lethal effect was predicted to be as short as 10 days, with susceptibility differences for the different *Culicoides* species. Using deltamethrin treated nets, another study showed that the insecticide was 90–100% effective in the laboratory test, but this dropped to 13% in field conditions [19]. Despite these findings on mortality rates, reductions in midge population sizes, biting rate or arbovirus transmission as a result of insecticide application have been harder to demonstrate under field conditions [20, 21] and there has been no study of how control impacts might vary seasonally.

The two main modelling approaches for understanding seasonal patterns of *Culicoides* disease vectors are statistical correlative models [22–25] or mechanistic mathematical or simulation models [2, 3, 26–28]. Whilst the statistical models provide accurate descriptions for the datasets they are trained on, they are limited to the datasets and are not readily extendable to new locations without additional data (though this has been done and integrated into transmission models [24]). They provide little insight to the mechanisms underpinning environmental responses of systems. For example, Searle et al. [25] linked the considerable intra-specific variability in *Culicoides* phenology between sites and years to a number of environmental factors including temperature, host density and larval habitat (also concluded by [23]) but the biological basis for these links was uncertain. Furthermore, it is not usually possible to use a fitted statistical models for scenario testing, such as assessing control strategies (statistical models that do this often assume that there is no explicit feedback between control efforts and the vector population dynamics, i.e. a lack of mechanism). In contrast, mechanistic models provide greater flexibility, but are often difficult to parameterise or fail to predict the seasonal patterns of vector abundance adequately.

Though environmental drivers such as temperature, rainfall and humidity are known to affect the abundance patterns and life-cycle parameters of midges [12, 29], their impacts on midge population dynamics are not well understood and may be opposing and nonlinear (for example high temperatures speed up development but reduce survival rates [30]). Temperature and moisture responses have been quantified in reasonable detail for only a single North American vector species, *Culicoides sonorensis*, because it is one of the only *Culicoides* vector species amenable to laboratory colonisation and manipulation [31]. In northern Europe, transmission of midge-borne viruses, namely bluetongue virus and Schmallenberg virus, involves multiple *Culicoides* species, from both the Obsoletus group (*C. obsoletus*, *C. scoticus*, *C. dewulfi* and *C. chiopterus*) and the Pulicaris group (*C. pulicaris* and *C. punctatus*), that are hard to separate morphologically [32] and colonise and are thus poorly characterised in terms of their ecological requirements. Though preferences for hosts and immature development sites will undoubtedly vary between the well-characterised North American vector species and the north European Obsoletus group species, it is possible that responses to temperature are similar, at least in functional form, between these similar-sized, temperate subgenus *Avaritia* species. We were interested in whether a mechanistic model of seasonal Obsoletus group populations, developed using the known temperature responses of the life-cycle of the North American vector species can predict UK abundance patterns and seasonal free vector periods of this taxa with the aim of targeting mitigation and prioritising life history knowledge gaps.

In this article we present a mechanistic simulation population model for *Culicoides* of the UK Obsoletus group species (comprised of the four species listed above), that incorporates the relationships between environment and demographic rates - the first of its kind for *Culicoides*. In this stage-structured model, we parameterise the environment-driven development rates using a mixture of laboratory and field data (fitting functional forms to known environment responses and back-fitting unknown parameters from abundance data). We demonstrate that the model accurately predicts UK dynamics. In particular, the model output exhibits the characteristic annual peaks in abundance in the spring and autumn, with similar lengths of annual adult presences compared to field data. Building upon this model framework, we investigate the efficiency of alternative timing and intensity of vector control. We demonstrate that targeted control may reduce the average vector abundance, but the timing and magnitude of the seasonal biannual midge population peaks may change and increase, depending on the control effort and timing.

## Methods

### Model structure

Contrasting previous studies [22, 25, 33], we have developed a new model framework for predicting the seasonal abundance of the Obsoletus group (defined above), which explicitly links environmental variation with life-history processes, such as development time, fecundity and mortality. These processes are modelled by functional forms that are parameterised by published laboratory and field environmental response experiments (see Additional file 1). The model is then back-fitted to abundance data to find the remaining unknown parameters (see Additional file 2) using an empirical dataset available from a prior study [23] within which the males and females of the four Obsoletus group species had not been identified separately. By modelling these species and sexes together, as prior studies have done (e.g. [23, 25]), we are assuming they have similar life-cycle responses to temperature and that density-dependence during the immature stages acts amongst as well as within these species and sexes. Although this is a simplification, the ability of the daily dataset to capture sudden phenological events like spring adult emergence outweighs the benefits of species-specific datasets that are temporally less well resolved. Furthermore, it has been demonstrated that Obsoletus group species can have similar patterns of abundance of female midges across a number of locations, including seasonal emergence and peaks of abundance [32], although it has been shown that male midges may show contrasting patterns [25].

**Fig. 1:**
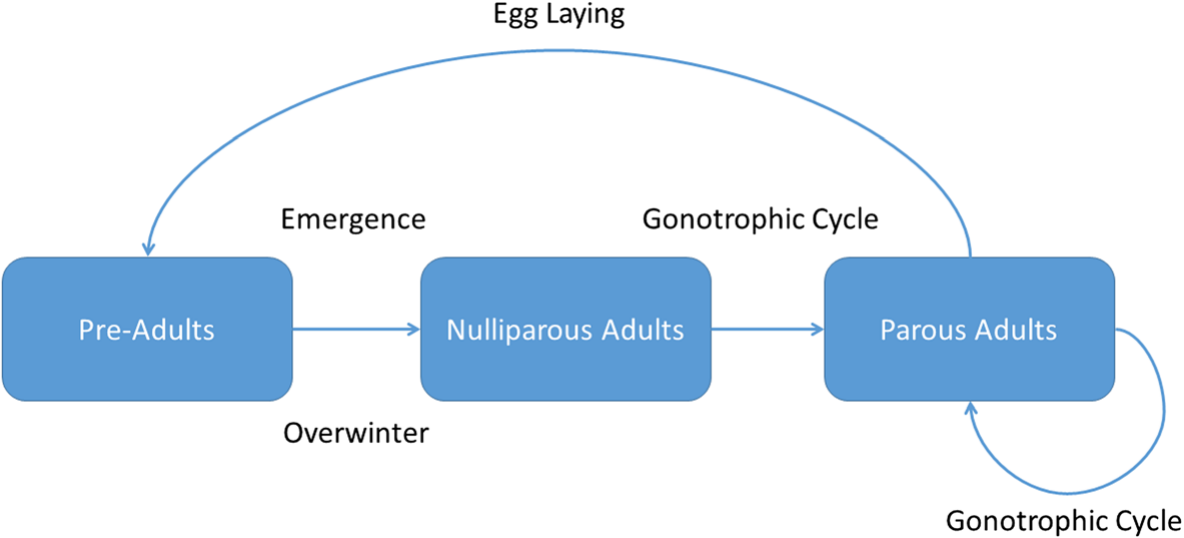
Schematic representation of the life-cycle of *Culicoides* spp. of the Obsoletus group

The model is stage-structured, modelling the distinct life-stages of the midge (see Fig. 1). For simplicity, and lack of parameter information, we have lumped together egg, larval and pupal stages into a pre-adult stage. The pupae emerge as nulliparous adults, whereby they require a blood meal after mating to complete their gonotrophic cycle. After this point, the adults are parous and they stay in this class until death. Overwintering (diapause) behaviour of *Culicoides* species is generally poorly understood, and depends on location and climate. In locations with mild winters, such as Mediterranean areas, populations may experience continuous development [34], but for locations which experience cold winters, such as northern European areas which we consider here, development is arrested. Field observations indicate that fourth-instar larvae are commonly present over winter [35, 36], and it is largely thought that temperature and/or photoperiod play an important role in determining when adult midges emerge from diapause from their immature stages in their semi-aquatic habitat [37–39]. Furthermore, it remains unclear if winter survival depends on larval instar or whether any development occurs throughout the winter period (and at what rate). Without further information, we make the assumption that eggs laid in the late autumn develop to fourth-instar larvae and arrest development prior to pupation. This assumption agrees with the data since adult spring emergence is highly synchronous [23, 25].

Within each class we define a number of cohorts, on top of which we define the age of the cohort (a scaled variable taking values between 0 and 1) and the number of individuals within a cohort (see later for how the latter is determined). The model runs on at daily timescale. From time *t* to time *t* + 1, each cohort is subjected to development and survival rates that fluctuate according to the current environmental conditions (air temperature, soil temperature and photoperiod). For example, in the pre-adult class cohorts at age 0 are newly laid eggs, at age 0.91 are pupating larvae and at age 1 are emerging nulliparous adults. As soon as cohort has an age greater than 1 it is moved to the next class (e.g. from pre-adult to nulliparous adult). If the number of individuals within a cohort goes below 1 then the cohort becomes extinct and is removed.

Many models that try to incorporate seasonality do so using a simplistic relationship between a single parameter (e.g. fecundity, say) that takes a sinusoidal functional form over the period of the season [40]. However, *Culicoides* fecundity, development and survival rates are highly sensitive to changes in environment, especially temperature [11]. Using a combination of laboratory for the North American vector, *C. sonorensis,* and field data for the Obsoletus group, we have fitted functional relationships between these stage-specific parameters and temperature (see Additional file 1). Thus, using Met Office air temperature data [23] and the Climate Hydrology and Ecology research Support System meteorology dataset for Great Britain (1961–2012) (CHESS) [41] land surface temperature as daily inputs, we obtain realistic seasonal parameters.

We implement development by a simple recursive equation. We denote the age of cohort *j* at developmental stage *i i* = pre ‐ adult, nulliparous adult or parous adult at day *t*by*A*_*j*_^*i*^(*t*). Then the development over a day is given by

$$ {A}_j^i\left( t+1\right)={A}_j^i(t)+{D}^i(t) $$

where *D* ^*i*^(*t*) is the daily development rate for development stage*i*, as defined in Additional file 1.

For simplicity, we assume an unrestricted availability and constant level of host abundance, from which adult midges may take a blood meal. This assumption is made in other models [5], although variation is expected between different spatial locations. Finally, we assume that larval classes undergo competition for resources [42]. We assume a daily competition rate proposed by [43], modified to incorporate multiple cohorts. This flexible functional form is given by

$$ {N}_j\left( t+1\right)= s(t){N}_j(t)\frac{1}{1+{\left( a{\displaystyle {\sum}_j}{N}_j(t)\right)}^b} $$

where, *N*_*j*_(*t*) is the number of individuals in cohort *j* at day *t*, *s*(*t*) is the density-independent pre-adult daily survival rate (see Additional file 1) and *a* and *b* are density-dependence coefficients that are location-specific. Since pre-adults inhabit semi-aquatic soil-type habitats, the daily survival and development rates, *s*(*t*) and *D*(*t*), depend on the soil temperature at the given location, given by CHESS [41].

To model diapause, we assume that pre-fourth-instar midges either survive at rate *s*(*t*) or develop at rate *D*(*t*). We make this assumption as only fourth-instar larvae are regularly found in winter soil samples [44] and the spring emergence of adult *Culicoides* is highly synchronised [23] (it can be shown that relaxing this assumption leads to less synchronous emergence). Pupae and fourth-instar larvae may develop at rate *D*(*t*) if *t* ∈ [*d*_start_, *d*_*end*_], where *d*_start_ and *d*_end_ are the day of diapause events at the start and end of the year, and are linked to photoperiod (see Parameter fitting).

### Simulations

The model simulations are coded in MATLAB^1^ and are freely available on GitHub [45]. The simulations are started on 1st January 2008 (to coincide with field observations) with 50 cohorts with 20 adults in each previously laying eggs, with numbers drawn from a Gaussian distribution, *N*(49.7, 11.1)_._ Initial pre-adult ages are also chosen from a Gaussian distribution, *N*(0.76, 0.1) (see Additional file 1). The model code is run once over a year and then restarted with the initial ages and abundances from the end of the model run. This step is to reduce transient effects, which typically only last for a single year.

### Parameter fitting

After the functional forms for survival, development and fecundity have been fitted (see Additional file 1) the model has a number of unknown parameters (density-dependence and over-wintering parameters) which we estimate by back-fitting the model to abundance data. We assume that the density-dependence parameters are site-specific, as these are likely to be related to local resource availability. There are two over-wintering parameters, which give the day at which diapause begins and ends. The over-wintering parameters are not site-specific, although differences in diapause will be observed in different locations depending on latitude (which defines the photoperiod).

**Fig. 2:**
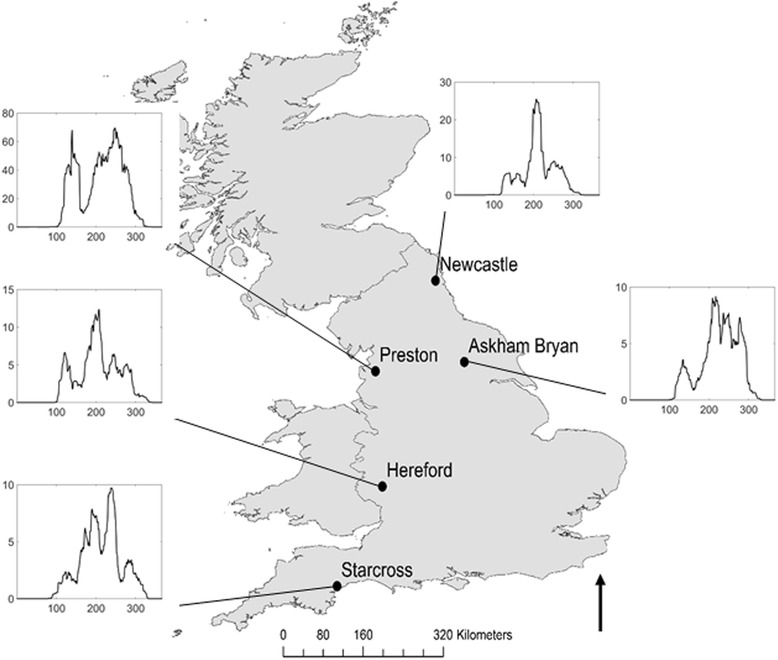
Locations of the five Rothamsted Suction-Trap sites where specimens of the Obsoletus group were collected. Inlays depict combined male and female Obsoletus group daily abundance data, smoothed using a 7-day moving window. Further details on the collection methodology and species identification can be found in [23] and [46]

We fit the model to male and female Obsoletus group, which comprises here of *C. obsoletus* (Meigen), *C. scoticus* Downes & Kettle, *C. dewulfi* Goetghebeur and *C. chiopterus* (Meigen) data from 12.2 metre Rothamsted Insect Survey suction-traps [46] sampled daily at 5 locations throughout the UK for 2008 (see Fig. 2) [23]. Since the trap data are a scaled representation of actual midge abundance, we apply a global scaling factor [5] to the midge abundance which is simultaneously fitted alongside the other parameters. We use a two-step process for fitting the model: a deterministic least squares fitting to give initial estimates for the model parameters, followed a stochastic approximate Bayesian computation (ABC) [47–50] using priors informed by the least squares fitting (see Additional file 2). This method is well-suited for fitting stochastic models [51].

## Results

### Population dynamics

**Fig. 3:**
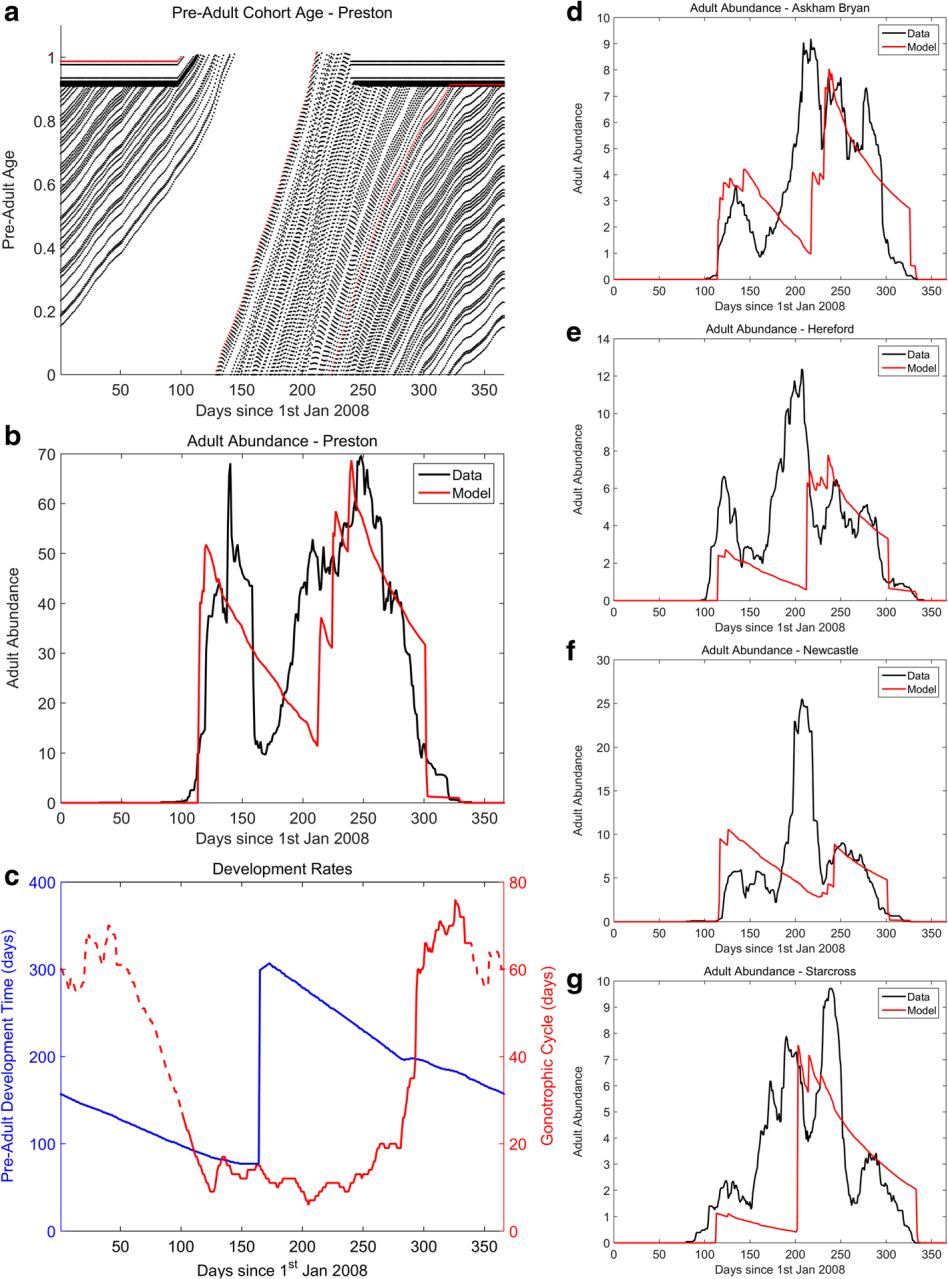
Fitted model output. In plots **a**-**c** we show the output of the model for the Preston location (see Fig. 2). In **a** the pre-adult cohort ages are plotted over time. The *red* line indicates the first cohort of the year to develop into adults. In **b** the smoothed (1 week moving average) total number of adults (nulliparous and parous) as predicted by the model (in *red*) and the corresponding field data [23] (in *black*) are plotted. In **c** the pre-adult development time (blue) and adult gonotrophic cycle length (*red*) are plotted, where the dashed lines indicate when the adults are not present (vector-free period). In plots **d**-**g** the adult abundances from the model output (*red*) and data (*black*) at Askham Bryan, Hereford, Newcastle and Starcross, respectively, are shown. All model parameters are given in Additional file 2: Table S2

In Fig. 3a-c we plot the fitted predicted seasonal abundance for the Preston location, which corresponds to the most abundant location. We first describe the mechanisms which lead to these seasonal patterns of abundance (also see Additional file 2: Figures S2 and S5).

In Fig. 3a we plot the age of each pre-adult cohort over time. At the start of the year (days 0–90), the plot shows that there is slow pre-adult aging up until the 4th larval instar, where development is assumed to arrest. In the spring (days 90–125), larval classes cease diapause and continue development at an increasing rate, as soil temperatures increase (also see Fig. 3c). This leads to new nulliparous adults emerging, with the adult numbers building up rapidly as large numbers of over-wintering pre-adults emerge (days 105–115, Fig. 3b). As adult midges develop, they begin to lay eggs, which result in new cohorts of pre-adults created at approximately 130 days after 1st January 2008, in mid-May. During the summer months (days 175–220), pre-adult development is markedly faster than in winter/spring, resulting in shorter generation times. As the autumn approaches, and climate conditions are less favourable, pre-adult development begins to arrest for fourth-instar larvae (day 225, mid-August; also see Fig. 3c), whilst earlier life-stages continue to develop at a decreased rate. By late winter, the development of pre-adult stages has markedly reduced (day 300 onwards).

In Fig. 3b we plot the predicted total adult abundances (nulliparous and parous) over time (in red) and the corresponding *Culicoides* catch data [23] (in black). It can be clearly seen that the model closely predicts the seasonal emergence and disappearance of adult *Culicoides*. Furthermore, the model also accurately predicts the timings and abundance of the two peak abundances in the spring and autumn. However, the model does less well in predicting the timing of the trough. This may be due to other environmental drivers (e.g. rainfall, wind or immature habitat drying out), which may have an effect on abundance and trapping efficiency [23].

By examining the pre-adult development and adult abundance, the mechanism of the adult biannual peaks can be determined. The sharp synchronous emergence of adult *Culicoides* is due to over-wintering pre-adult stages that emerge closely together, which in turn is caused by pre-adults arresting development at the fourth-instar larval stage. This results in large numbers of eggs being laid in a synchronous fashion, which then develop into adults, producing the sustained second peak, as the adults complete a number of gonotrophic cycles (Fig. 3c). It can be clearly seen by following the first cohort of pre-adults (Fig. 3a - red dots) that there are only two adult generations per year; the third potential generation is unable to develop sufficiently quickly and over-winters as pre-adults. A third generation may be possible in favourable conditions, that is, a warm early spring, which continues through to late autumn. It remains to be shown if this is possible in the UK, since the diapause parameters also restrict early adult emergence.

We have used our model to predict the Obsoletus group midge abundance patterns, contrasting it with geographical varying, daily data (Fig. 3b, d-g). Our model fits the data well for most sites (Askham Bryan, Hereford, Preston and Starcross), but does not agree well for the Newcastle site, for which the data suggest a unimodal pattern of abundance as opposed to the signature bimodal pattern.

### Population control

Our mechanistic modelling framework allows us to model the impact of control measures on the seasonal abundance of biting midges. No single *Culicoides* vector control method is in routine field operation [52]. Our approach is motivated by recent experimental evidence of field efficacy of some chemical control methods, focussing on insecticide control that targets host-seeking adult midges [18, 19, 53, 54] as being the best control option currently available [54]. In particular, insecticide screening of animals has high potential value in the early stages of an epidemic and during transport of susceptible animals to prevent further spread [52]. We assume that host-seeking adult midges will visit an insecticide treated trap and that the trap has a probability of killing the adult, such that

$$ \mathrm{Control}\ \mathrm{Efficiency} = \mathrm{Fraction}\ \mathrm{of}\ \mathrm{midges}\ \mathrm{visiting}\ \mathrm{n}\mathrm{e}\mathrm{t}\ \mathrm{or}\ \mathrm{host} \times \mathrm{Probability}\ \mathrm{of}\ \mathrm{death} $$

Thus, if all host-seeking adult midges visit the insecticide treated trap, then the control efficiency is simply the probability the midge will die from insecticide contact. Field studies with deltamethrin-treated nets controlling *Culicoides* species in Spain showed that mortality (probability of death) was approximately 13% [19]. In a field study on sheep applied with insecticide the short-term mortality rate was 49% [18]. However, it was not possible to ascertain from these studies what fraction of the host-seeking midge population visited the trap or host, as host-seeking midges may find alternative hosts to feed upon and therefore avoid the trap. We overcome this difficulty by treating this parameter implicitly by rolling it into the control efficiency according to the above relationship. In our analysis we consider two scenarios: constant control, where the insecticide is used and equally effective throughout the year; and where the insecticide is applied at a constant level for a set period of time at variable times of the year. These analyses allow us to firstly understand the overall impact of the insecticide and then to unveil the most efficient time to use the insecticide.

**Fig. 4:**
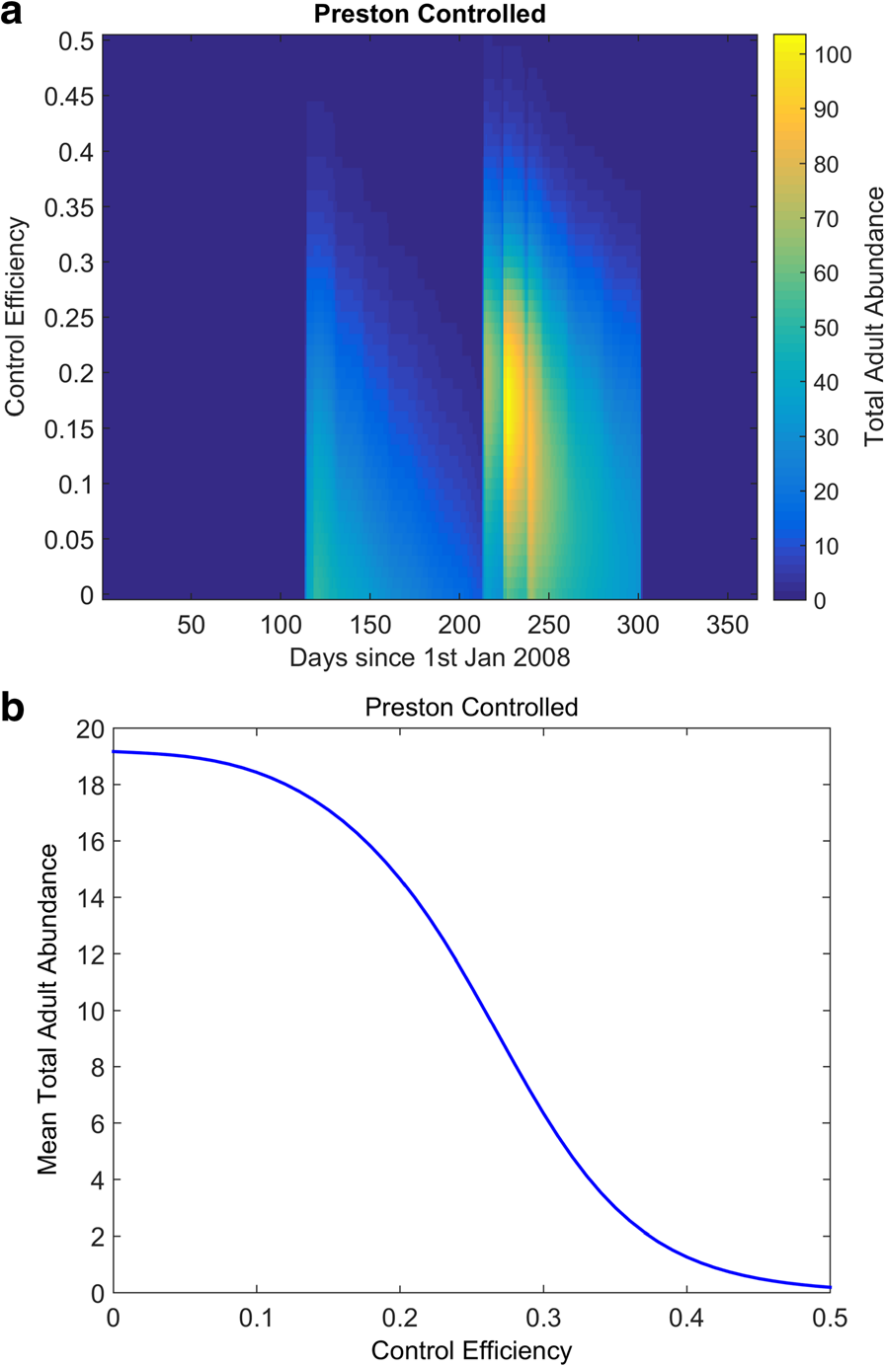
The effects of varying the control efficiency on the adult midge population at Preston. The midge model is run with varying values of control efficiency and the total annual adult (nulliparous and parous) population is plotted, using the graduated colour scale (**a**). The transect corresponding to when the control efficiency is zero corresponds to the *red* line in Fig. 3b. In **b**, the average of the total adult abundances for each value of control efficiency is shown

We begin with the constant control scenario in Fig. 4. In Fig. 4a, the colours denote total midge abundance. When control efficiency is zero (i.e. no control strategy is applied), we may draw a transect through the colours and the dynamics are equivalent to those presented in Fig. 3b. Figure 4b depicts the average total adult midge abundance for varying control efficiencies.

These plots show that for significantly high control efficiencies, we may eradicate the midge population, since both nulliparous and parous adult Obsoletus group midges require a blood meal to complete their gonotrophic cycle. Note that this may not hold for autogenous *Culicoides* species where nulliparous adults do not require a blood meal such as *Culicoides impunctatus*. In reality, it may not be possible to achieve such high control efficiencies for non-autogenous species like *C. obsoletus*. For example, if the desired control efficiency is 50% (which would ensure that almost all midges are eradicated) and the probability of death is very efficient at 0.75, then 2/3 of the local midge population must visit the trap. For low control efficiencies, there is a small reduction of the average midge abundance. For intermediate control efficiencies the average annual midge abundance markedly reduces. However, the peak autumn abundance is greatly increased under intermediate efficiency, year-round control, as depicted by the red areas Fig. 4a. This is because of pre-adult density-dependence relief caused by the decrease in population in the spring peak [55]. As a consequence, although the control strategy has had a desirable effect on the whole, the increase in peak autumn abundance may have dramatic consequences for R_0_ and the prevalence of midge-borne diseases. These temporal results suggest that applying a control strategy throughout the year may not yield the most ideal control and that targeting specific times of the year may be more beneficial, and cost-effective. Thus, we ask, when should we concentrate our effects for adult insecticide control?

**Fig. 5:**
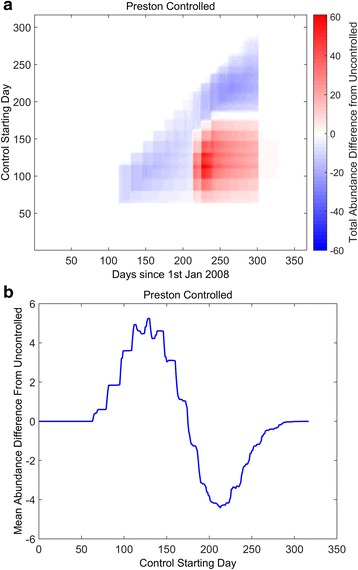
Effects of a window of insecticide control. In contrast to Fig. 4, the control is not constant throughout the year, but instead a window of insecticide control is applied at different times of the year. It is assumed that within the window the control efficiency is 0.2, and 0 elsewhere. The length of the window is fixed to 50 days and the starting day of the window varies, plotting the difference in observed total adult abundances to the uncontrolled equivalent (**a**). Areas in *white* show no differences in abundance; *red* show greater abundance; *blue* show less abundance, compared to the uncontrolled population. In **b**, the mean differences over the year for varying starting times are plotted. The control strategy is run for one year only

In Fig. 5 we consider a scenario where there is a window of control, in contrast to a constant control strategy (cf. Fig. 4). Here, the insecticide is present at a constant level for only a limited time in the year and we varying the starting day at which the window starts. Pour on solutions of deltamethrin have been shown to remain active even after 21 days post inoculation on sheep wool, and up to 35 days on cattle and horse hair [56, 57]. Without loss of generality, we assume a best case scenario where the insecticide is fully lethal for 50 days, after which it is neutralised. We plot the differences observed in the controlled scenario compared to the uncontrolled scenario. The figure shows intuitively that for windows at either the beginning or end of the year there is no effect on the population dynamics (Fig. 5a - white horizontal transects; Fig. 5b - zero difference), since there are little to no adult *C. obsoletus* midges present (midge populations over-winter as pre-adults). When the window centres over the spring abundance peak (approximately 100th starting day), the spring peak is reduced (Fig. 5a, lower left blue area). However, the knock-on effect is a markedly increased autumn peak (Fig. 5a, lower right red area). Furthermore, the mean abundance throughout the year also increases (Fig. 5b, peak). In contrast, for control windows centring over the autumn midge peak in abundance (approximately 200th starting day), there is obviously no effect on the prior spring population (Fig. 5a, upper left white area). In contrast, windows centring over the autumn peak markedly reduce the annual midge population (Fig. 5a, upper right blue area).

**Fig. 6:**
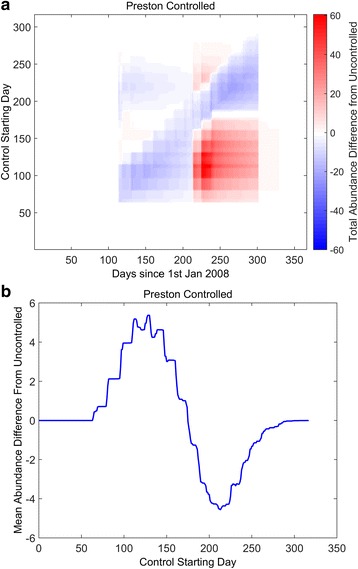
Effects of repeated, year on year, window of insecticide control. In contrast to Fig. 5, the window of insecticide control is repeated year on year. It is assumed that within the window the control efficiency is 0.2, and 0 elsewhere. The length of the window is fixed to 50 days and the starting day of the window varies, plotting the difference in observed total adult abundances to the uncontrolled equivalent (**a**). Areas in *white* show no differences in abundance; *red* show greater abundance; *blue* show less abundance, compared to the uncontrolled population. In **b**, the mean differences over the year for varying starting times are plotted. The control strategy is run for one year only

It can be shown (see Fig. 6a - top left blue area) that repeated, year on year, control strategies that focus on the autumn peak not only reduce the autumn midge population, but also reduce the subsequent spring population, since fewer individuals enter diapause. For late autumn windows of control, the late autumn midge peak is greatly reduced, as well as the subsequent spring population. However, due to reduced density-dependence, the late summer midge population increases in the subsequent year (Fig. 6a, upper right red area), although on average, the annual midge population is reduced (Fig. 6b, positive gradient after trough).

Thus, we conclude from these figures, that control efforts should concentrate on reducing the autumn peak, as this will reduce the midge population at the peak, as well as on average (Fig. 6b, trough). However, the precise timing is crucial for effective control, especially if the strategy is used year upon year.

## Discussion

In the paper we have derived a mechanistic stage-structured simulation model, which accurately predicts the dynamics of Obsoletus group over a range of UK sites. The model successfully predicts the early spring emergence, winter decline and biannual peaks of abundance, which are important features of midge seasonality in northern Europe [33], and are key determinants of the risk of establishment (R_0_ [2, 3, 5]) and spread of midge-borne diseases [58, 59]. Our model predicts that the spring and autumn peaks of adult abundance correspond to two generations per year (bivoltine), which we can observe in the pre-adult development data (Fig. 3). However, other studies have concluded that three generations (trivoltine) may be possible, based on the observation of the number of peaks in catch data [60, 61]. However, for the scenarios considered in this study, we find trivoltinism to be unlikely, although further research into how dietary media and other environmental factors influence voltinism should be considered, as discussed by Holmes & Boorman [62].

Whilst our model successfully predicts the seasonal patterns of abundance for Askham Bryan, Hereford, Preston and Starcross, which exhibit the characteristic bimodal pattern of abundance [23], our model fits the unimodal pattern of Newcastle less well. This suggests that our model is not sufficiently flexible to be able to predict unimodal dynamics. However, there are a number of caveats that should be taken into account in understanding the data. *Culicoides* abundance patterns are highly variable [24] and can be influenced by multiple environmental drivers [23, 25, 33], with trapping efficiency significantly dropping in sub-optimal climate conditions. Furthermore, the species comprising the Obsoletus group may exhibit different seasonal abundance patterns, which, in theory, could lead to unimodal patterns of the group [23].

Since the model accurately predicts the spring emergence and autumn decline of adult midges, it provides a framework for estimating the seasonal vector-free period, which is important for livestock movement and timing of vaccination in epidemic zones. Importantly, Searle et al. [25] demonstrated that active surveillance of haematophagous female *Culicoides* vector populations cannot currently be replaced using remote environmental models of abundance since there are complex biological interactions at play which determine annual patterns of abundance. Combining accurate models of vector dynamics, coupled with realistic disease models, will give greater predictive abilities and hence mitigation strategies. Whilst some epidemiological models focus on predicting vector-borne diseases, such as BTV, many do not explicitly model the seasonal population dynamics [59], or assume simplistic or correlative models [2, 3, 26, 28, 58, 63]. However, the complex mechanistic vector dynamics are important as they affect disease seasonality and persistence between years [5, 64], and are affected by environmental variability.

As our novel model is mechanistic, we have been able to hypothesise how control strategies, namely adult host-seeking insecticide traps, might impact on the insect vector population. We have shown that control strategies may either successfully reduce the vector population, or deleteriously increase the vector population. This counterintuitive result depends on the timing of control strategies, where we predict that it advantageous to target control strategies on the autumn midge peak, as this has the greatest effect on reducing the vector population. Whether or not the controlled midge population increases preceding control efforts is likely to depend on the functional form and strength of density-dependence [55, 65, 66]. Though high densities are known to reduce immature survival in North American species [42], nothing is known about density-dependence in Palaearctic *Culicoides*. Furthermore, field studies on the impact of insecticides on midge abundance are few and far between, with most studies focusing on control efficiency using baited traps (e.g. [18]). However, Satta et al. [67] conducted an area-wide application of a pyrethroid insecticide and found that treated areas did not have significantly fewer *Culicoides* midges, compared to the untreated areas. It is unclear whether the poor performance of disinfestation through insecticides [67] is because insecticide application is untargeted (relative to the preferred habitats of *Culicoides* vectors) and local and transient in nature of the application or due to density-dependent effects. We would urge that further experimental studies into this area are conducted.

More generally, the model has highlighted several areas where little is known about Palaearctic *Culicoides* biology and ecology. We have made assumptions about these in the model, but it is likely that our results are dependent on these assumptions. They include:

### (i) Diapause or quiescence

It is known that temperature plays an important role in the development of diapausing *Culicoides* larvae [37], the precise stages that survive the winter are unknown or whether any development occurs over the winter period. For example, do short-term increased temperatures incite development over winter in some individuals (quiescence rather than true diapause)? Also, does mortality act differentially across different life-stages in diapause? Changing the diapause assumptions demonstrate (not shown here) that they play an important factor in the timing of the midge spring peak, which also has consequences for the remaining dynamics in the year.

### (ii) Fecundity

In our model, it is assumed that fecundity remains constant throughout the lifetime of the adult female midge. In reality, it is more likely that fecundity (egg development time and clutch size) is dependent on the age of the female midge, temperatures experienced during development and whether a blood meal has been taken, especially for autogenous species [68], although few data exist to corroborate this.

### (iii) Other environmental drivers

Our model considers air and soil temperature to be the main driver of seasonality, but it stands to reason that other environmental drivers, especially rainfall, soil moisture and humidity, are import determinants too [25, 69, 70]. However, teasing apart the relationship between actual midge abundance and trap data may be problematic since rainfall and wind speed, for example, influence flight behaviour [71]. In future model developments, we aim to include other environmental drivers. For example, it is likely that soil moisture plays an import part in the development of pre-adult life-stages [72], as well as resource competition.

### (iv) Insecticide decay

For simplicity, we have assumed that the insecticide lethality remains at constant levels or is constant within a given window. However, in practice, insecticide treatment efficacy is likely to decay over time [73], and may depend on the environment that the hosts inhabit [74]. This, combined with the frequency of application, suggests that insecticide lethality is likely to fluctuate in time and therefore so does their control efficacy. We aim to investigate this fluctuation, and make recommendations for treatment schedules.

### (v) Scaling between trap data and population abundance

A key determinant of control efficiency is the fraction of the surrounding population of midges that is impacted by control by visiting traps. It is also a critical parameter in translating data from traps (and possibly for different types of traps e.g. suction or light traps) into vector-host ratios in spatial R_0_ models of disease establishment [5]. Furthermore, in this paper we have assumed that there is a scaling parameter between catch data and population abundance. These facts beg the question of how do catch data scale into population density and how does this affect our inference of disease model predictions?

### (vi) Non-constant host abundance

In each of the sites in this study, we have made the assumption that hosts are equally abundant for midges to take their blood meal. In reality, host abundance plays an important role in determining *Culicoides* abundance [5, 25]. In future studies, the local densities of hosts and the way *Culicoides* abundance scales with the densities of different host types should be accounted for.

### (vii) Trivoltinism

For the scenarios we have considered here, the model predicts that the midge populations have two complete generations. It has been long suspected that climate conditions could incite a third generation, which would not only have an import implication for midge seasonal abundance, but also disease. In future developments, we will aim to understand under which climate conditions this could occur.

Addressing the above knowledge gaps will further our understanding of vector *Culicoides* ecology and enable greater accuracy for predictive models of disease control.

A robust test of our mechanistic model framework is whether the timing of adult emergence in spring and disappearance in autumn can be accurately predicted, preferably to within one to a few days. We thus opted to confront the model with geographical varying, daily patterns in adult abundance patterns from a suction trap network in the UK. The few species-level adult surveillance datasets that exist across Europe tend to be available at lower monthly or weekly temporal resolutions, are not publicly available and still do not distinguish between *C. obsoletus* and *C. scoticus* [32, 75]. While male members of the subgenus *Avaritia* in western Europe can be identified reliably based upon marked differences in their genitalia, the routine morphological identification of the females that are important in transmission is less straightforward (see [76] and references therein). This has led to the development of PCR-based identification assays for the species of the subgenus *Avaritia*, particularly required for distinguishing between two widespread and abundant species, *C. obsoletus* (*s.s.*) and *C. scoticus*. However, these techniques are still costly for application to large-scale datasets like the one required here and are less effective on older samples. In the long term, the use of quantitative real-time PCR assays for identification of pooled specimens may address these methodological issues [77] but are yet to be fully optimised [78].

The population model developed here is at the group-level, the most commonly available data type in Europe. As such, there would be a great benefit to predict the dynamics of individual species rather than treating these species as a group, and to predicting abundance in other locations throughout Europe, as well as other species of biting midge. Clearly, extending our model would require more midge data with a high temporal and taxonomic resolution so that the model can be calibrated and validated, but this is likely to be labour intensive [32].

## Conclusions

Seasonal peaks and troughs of abundance of biting midges are key determinants of the risk of establishment and spread of midge-borne diseases. We have developed a mechanistic model which reproduces these characteristic seasonal dynamics. Our model suggests that the peaks correspond to two generations per year (bivoltine) are largely determined by pre-adult development. Furthermore, our model shows that control strategies should focus on reducing the autumn peak since the immature stages are released from density-dependence regulation. We conclude that detailed modelling of *Culicoides* biting midges will help to better develop control strategies and predictions of disease outbreaks.

## Additional files

Additional file 1: Parameter curves for development stages as functions of temperature. (PDF 254 kb)

Additional file 2: Parameter fitting. (PDF 611 kb)

## Footnotes

1 © 2015 The MathWorks, Inc. MATLAB and Simulink are registered trademarks of The MathWorks, Inc.
