# Supplementary material for: Mechanistic model for predicting the seasonal abundance of Culicoides biting midges and the impacts of insecticide control
Source: Parasit Vectors. 2017 Mar 27;10:162. doi: 10.1186/s13071-017-2097-5 (PMC5369195; doi:10.1186/s13071-017-2097-5)
Supplement: Supplementary file 1 — Parameter curves for development stages as functions of temperature. (PDF 254 kb) [file 13071_2017_2097_MOESM1_ESM.pdf]

Additional file 1: Parameter curves for development stages as functions of temperature

Here we provide the details of fitted parameter curves for each of the development stages as functions of temperature. Further discussion on life-cycle parameters can be found in Searle et al. [1]. It should be noted that although the model describes the population dynamics for the Obsoletus group described in the main text, we use lab-derived survival and development data for the North American vector, *Culicoides sonorensis*, since it is the only temperate species that has been colonised for which such data are available. Only the egg clutch size parameter estimation is derived from the Obsoletus group. Though we do not expect that development and survival parameterisation from the Obsoletus group data would dramatically change our findings, it would be desirable to test this assumption in the future.

Pre-Adult Development

The relationship between development rate from egg to nulliparous adult and temperature was derived for *C. variipennis sonorensis* taken from EJ Wittmann [2]. There was higher variance in duration of development across females at lower temperatures.

Table S1: Table reproduced from Chapter 2 of EJ Wittmann [2]. The table shows the development times of *C. variipennis sonorensis* eggs, larvae and pupae at a range of constant temperatures.

Temperature in (ºC)	Day of first emergence of larval instars				Duration to emergence of			
					pupae		Adults	
	1st	2nd	3rd	4th	Mean	s.e.	Mean	s.e.
12.5	11	12	40	53	132.2	0.5	142.2	0.59
15	5	7	21	25	70.6	0.13	77.2	0.14
20	4	6	7	9	35.1	0.06	38.3	0.06
25	2	4	6	9	24.4	0.07	26.5	0.08
30	2	3	5	7	13.7	0.02	15.2	0.02
35	1	2	3	4	12.9	0.04	14.3	0.04

Development to the pupal stage took on average 91.4% of this egg to nulliparous adult development time. We keep a check of whether larvae have reached this stage in the model, since temperate *Culicoides* are thought to diapause in the fourth larval instar [3]. Whether this is a true diapause that responds to photoperiod or whether this is the stage that individuals tend to get to before cold temperatures arrest development over winter is not well understood.

From the data presented in Table S1 can be converted to a temperature dependent rate by taking the reciprocal of the duration. Using least squares regression, a functional form for the rate can be found (see Figure S1). The exponential curve provided a good fit for the data and suggests that development slows at low temperatures. An alternative fit could be a linear fit ($y=0.003x-0.032$, $R^2=0.9705$), which would suggest that there exists a developmental threshold around 10.7ºC. However, given the synchronous spring emergence of adult midges [4, 5], over-winter arrested development and survival of early instar larvae seems unlikely. Therefore, either early instar larvae continue development or perish over winter. Simulations suggest (not shown here) that the latter results in small spring peak abundances, which does not fit with the data

[4]. Therefore, we make the former assumption and use the functional form as shown in Figure S1.

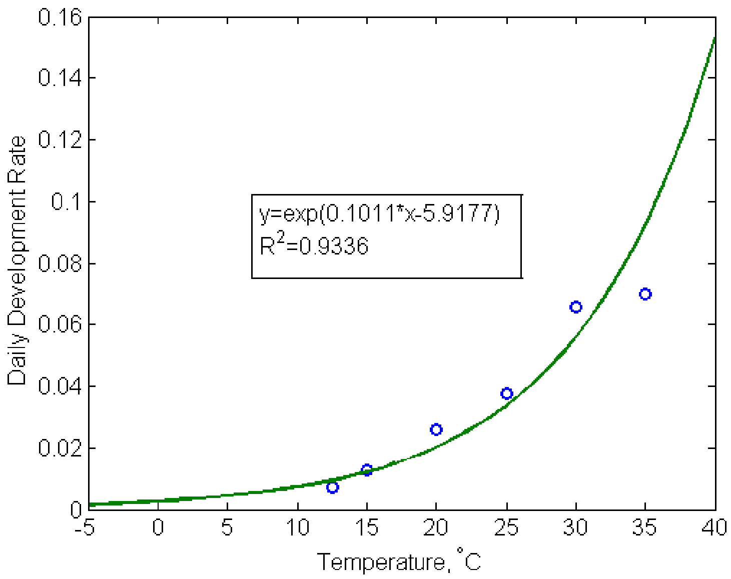

Figure S1: Fitted curve to the pre-adult *Culicoides* development rate data. All data come from EJ Wittmann [2], where the development rate (measured in day^{-1}) is defined by the reciprocal of the development time.

Pre-Adult Survival

EJ Wittmann [2] also examined survival through the larval stage to adult emergence in *C. variipennis sonorensis* at different temperature treatments. Here, the proportion of surviving pre-adult population is measured across a range of temperatures. Note that the survival probability is only measured from egg to adult, rather than the transition through each development stage. Due to the lack of this information, we make the simplifying assumption that survival acts uniformly across each development stage, although this is unlikely to be true. Under this assumption, it follows that the survival probability from egg to adult ($\hat{S}$) is simply the daily survival probability (s) raised to the power of the development time (d), that is, $\hat{S} = s^d$. Thus, we may calculate the temperature dependent daily survival probability, since the duration is given in Table S1 and rearranging our equation we have $s = \hat{S}^{1/d}$.

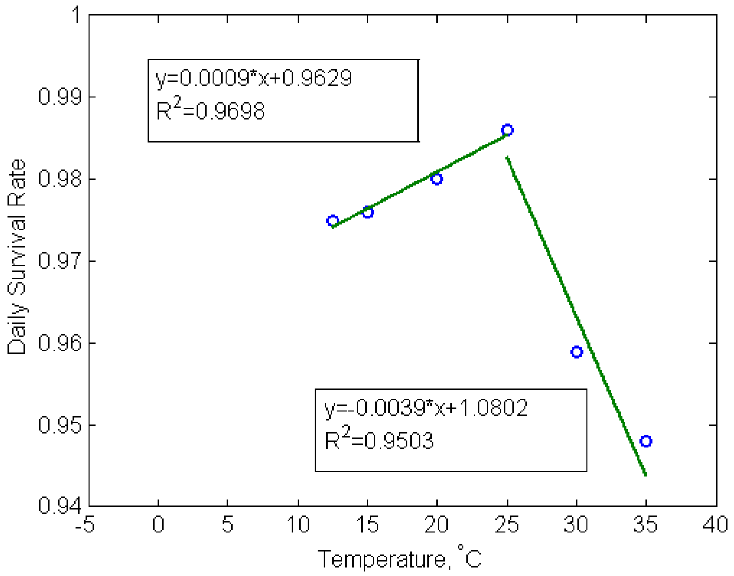

Figure S2: Fitted curves for the daily survival rates of pre-adult *Culicoides obsoletus* group midges. All data come from EJ Wittmann [2], with conversions as per the main text. The regression is split into two, to represent increasing and decreasing pre-adult survival as temperature increases.

Figure S2 shows that daily survival rates were high at low to medium temperatures, and optimal at 25ºC, and then drop off rapidly with temperature. We fitted linear curves to the data before and after the optimum, and fixed survival rates above 35ºC and below 12.5ºC to the respective set values.

It should be noted that other functional forms, rather than two straight lines, could be fitted to the data. However, without greater data to support the trend, such attempts are likely to result in overfitting. Thus, we have chosen the simplest model. Beyond the temperature ranges of the experiments, we assume constant survival rates.

Adult Development

BA Mullens and FR Holbrook [6] studied the relationship between temperature (T) and time needed (in days) for more than 80% of females to mature oocytes in *C. variipennis sonorensis* (d). They showed that this relationship has the fitted functional form

$$d = -1.98 + 0.07217T + 2516.65T^{-2},$$

where R^2=0.998. Thus, the daily development rate for an adult gonotrophic cycle is given by the reciprocal of the above equation ($1/d$). We plot this relationship in Figure S3 below.

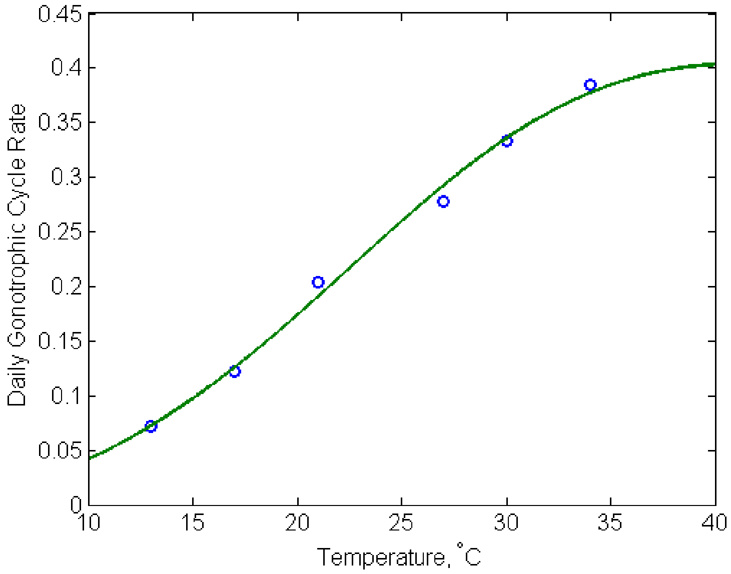

Figure S3: The daily adult gonotrophic cycle rate (days^{-1}) plotted over varying temperatures. All data and fitting come from BA Mullens and FR Holbrook [6].

In our model, it is assumed that the gonotrophic cycle remains constant and temperature-dependent throughout the lifetime of the adult female midge. In reality, in is more likely that fecundity (development time and clutch size) is dependent on the age of the female, although no data exists to corroborate this.

Adult Survival

The literature has conflicting evidence on the effect of temperature on survival through the adult stage. Wellby et al. [7] found that survival rates at 10, 15, 20ºC were very similar with between 80-90% of individuals still being alive after 14 days of adult hood. By contrast at 25ºC, 40% of individuals were alive after 14 days and at 28ºC, 10% were alive after 10 days.

These data are discussed again in Mellor et al. [8] where we find that comparison of daily survival rates (inferred from adult stage survival raised to the power of 1/15 as above) between temperature treatments is only really possible after 2 weeks of adulthood since this is the only time point up to which insects were consistently examined across treatments.

Wittmann et al. [9] found that the longevity of adult *C. sonorensis* decreased significantly as temperature increased from 15 to 30ºC, but was not significantly affected by saturation deficit or relative humidity though the interaction between temperature and saturation deficit was significant. That is, at low temperatures, survival was greater at high relative humidity (85%) versus low (40-75%). At high temperatures, the impact of relative humidity was reversed. She modeled this with a different hazard function (instant risk of death) for high and low relative humidity.

Hunt et al. [10] studied the effects of several environmental factors on mortality of adult *C. sonorensis* in the laboratory. They found that daily mortality rates significantly increased when adults were maintained at an elevated constant temperature (26° C).

To handle this variability, we amalgamate the data from these studies and fit a single cubic curve, as shown in Figure S4. This shows that at low temperatures, there is little change in the daily survival probability of adult midges. However, increasing temperature has a dramatic reduction in survival.

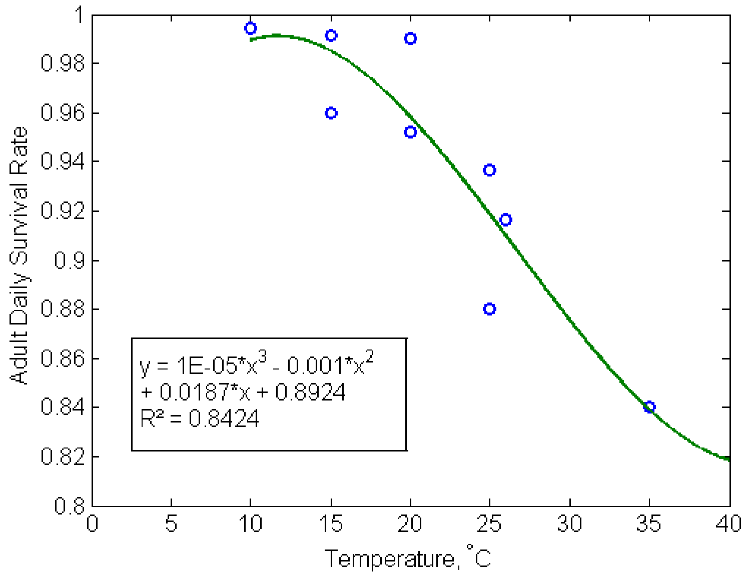

Figure S4: The daily adult survival rate plotted over varying temperatures. The data is an amalgamation of from three datasets [8, 9, 10] on *Culicoides sonorensis*.

Adult Clutch Size

We took the average and standard deviation of clutch sizes for *C. obsoletus* (51.40 ± 7.30) and *C. chiopterus* (48.00 ± 8.30) given in MW Service [11] to give a group mean and standard deviation of 49.7 ± 11.054. At each time step, we drew from a normal distribution with the group mean and standard deviation to define the number of eggs laid for each laying cohort.

Laboratory experiments on *C. variipennis sonorensis* indicate very little variation in clutch size between temperature treatments except when temperatures drop very low (around 12°C for this species), where upon only a handful of eggs were produced per female with very few females ovipositing at all (there was also evidence of resorption of eggs at low temperatures) [6]. Thus, we assume that the Obsoletus group lay only 5 eggs at temperatures below 12°C.

References

1. Purse BV, Carpenter S, Venter GJ, Bellis G, Mullens BA. Bionomics of temperate and tropical *Culicoides* midges: knowledge gaps and consequences for transmission of *Culicoides*-borne viruses. Annu Rev Entomol. 2015;60 1:373-92.
2. Wittmann EJ: Temperature and the transmission of arboviruses by *Culicoides* biting midges. University of Bristol; 2000.
3. Kettle D, Lawson J. The early stages of British biting midges *Culicoides latreille* (Diptera: Ceratopogonidae) and allied genera. Bull Entomol Res. 1952;43 03:421-67.
4. Sanders CJ, Shortall CR, Gubbins S, Burgin L, Gloster J, Harrington R, et al. Influence of season and meteorological parameters on flight activity of *Culicoides* biting midges. J Appl Ecol. 2011;48 6:1355-64.

5. Searle KR, Barber J, Stubbins F, Labuschagne K, Carpenter S, Butler A, et al. Environmental drivers of *Culicoides* phenology: how important is species-specific variation when determining disease policy? PLoS One. 2014;9 11:e111876.
6. Mullens BA, Holbrook FR. Temperature effects on the gonotrophic cycle of *Culicoides variipennis* (Diptera: Ceratopogonidae). J Am Mosq Control Assoc. 1991;7 4:588-91.
7. Wellby MP, Baylis M, Rawlings P, Mellor PS. Effect of temperature on survival and rate of virogenesis of African horse sickness virus in *Culicoides variipennis* sonorensis (Diptera: Ceratopogonidae) and its significance in relation to the epidemiology of the disease. Bull Entomol Res. 1996;86 06:715.
8. Mellor PS, Rawlings P, Baylis M, Wellby MP. Effect of temperature on African horse sickness virus infection in *Culicoides*. Arch Virol. 1998:155-63.
9. Wittmann EJ, Mellor PS, Baylis M. Effect of temperature on the transmission of orbiviruses by the biting midge, *Culicoides sonorensis*. Med Vet Entomol. 2002;16 2:147-56.
10. Hunt GJ, Tabachnick WJ, McKinnon CN. Environmental factors affecting mortality of adult *Culicoides variipennis* (Diptera: Ceratopogonidae) in the laboratory. J Am Mosq Control Assoc. 1989;5 3:387-91.
11. Service MW. Light-trap catches of *Culicoides* spp. (Dipt., Ceratopogonidae) from southern England. Bull Entomol Res. 1969;59 02:317.
