# Supplementary material for: Mechanistic model for predicting the seasonal abundance of Culicoides biting midges and the impacts of insecticide control
Source: Parasit Vectors. 2017 Mar 27;10:162. doi: 10.1186/s13071-017-2097-5 (PMC5369195; doi:10.1186/s13071-017-2097-5)
Supplement: Supplementary file 2 — Parameter fitting. (PDF 611 kb) [file 13071_2017_2097_MOESM2_ESM.pdf]

Additional file 2: Parameter fitting

Here we detail the methods and results of fitting the midge model to the time-series data. Firstly, we use a deterministic least squares fitting, which is then used to provide priors for an Approximate Bayesian Computation [1, 2, 3, 4] fitting.

For the fitting below, the temperature-dependent parameters (see Additional file 1) are fixed. Allowing these to be estimated in a Bayesian framework would have enabled us to test whether the functional form of these temperature relationships differed between the Obsoletus group and *C. variipennis sonorensis*, the estimation of these on top of the other parameters would have been prohibitive computationally. We only fit the model to parameters where there is no prior information, namely the density-dependence (a_i and b_i, corresponding to site i), diapause (d_{start} and d_{end}) and scaling factor (c) parameters. We assume that the density-dependence parameters are site specific, as these are likely to be related to local resource availability. There are two over-wintering parameters, which give the start and end of diapause. We assume that diapause is correlated to photoperiod, although the mechanisms of diapause in *Culicoides* are poorly understood. The over-wintering parameters are not site-specific, although differences in diapause timings will be observed in different locations depending on latitude, which defines the photoperiod (see Figure S1). The amount of daylight in a day is calculated by a planetary motion equation[1].

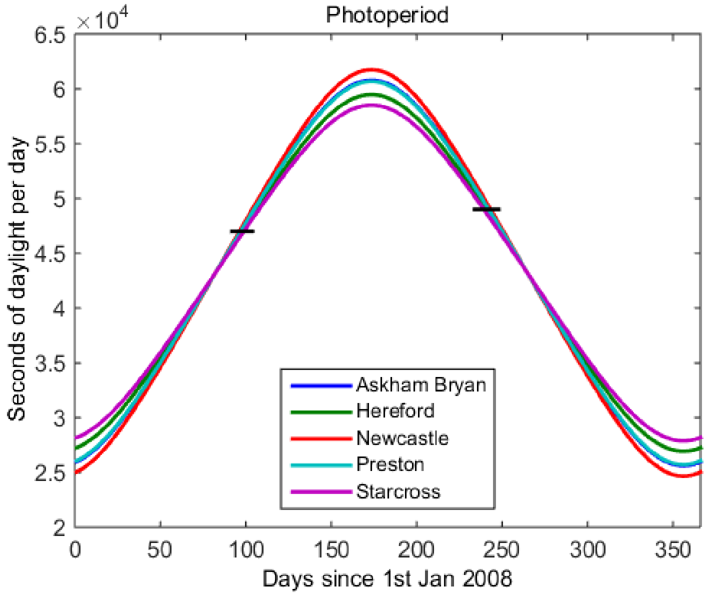

Figure S1: Calculated photoperiod for the five sites of interest. The diapause parameters, $\boldsymbol{d}_{\text{start}}$ and $\boldsymbol{d}_{\text{end}}$ (diapause at the start and end of the year), are defined by the daily amount of daylight. Thus, diapause acts at different times of the year, depending on the latitude of the site. These parameters are unknown and are fitted using the statistical methods described.

[1] http://www.gandraxa.com/length_of_day.xml

We fit the model to adult Obsoletus group data from daily suction traps [5] at five locations throughout the UK (see Figure 4 in the main text). Since the trap data is a scaled representation of actual Obsoletus group abundance, we apply a global scaling factor [6] to the Obsoletus group abundance which is simultaneously fitted alongside the other parameters.

Least Squares Fitting

Performing least squares fitting to stochastic models is inherently difficult due to the stochastic nature of the residual surface. Therefore, as a first step, we implement a deterministic version of the model for parameter fitting by choosing mean values for the parameters (e.g. the adult clutch size is taken to be the mean, 49.7 eggs).

The method we employ is a least squares minimisation where the sum of squares of the residual (difference between model output and data) is minimised. We use a constrained Nelder-Mead algorithm[2], implemented in MATLAB[3], to find the optimal parameter values. Initial estimates are given over a random wide bounded range, to militate against local optima and the relative uncertainty of the model parameters. We run 1000 optimisation runs and retain the best fit parameters, the result of which is shown in Figure S2 and Table S1.

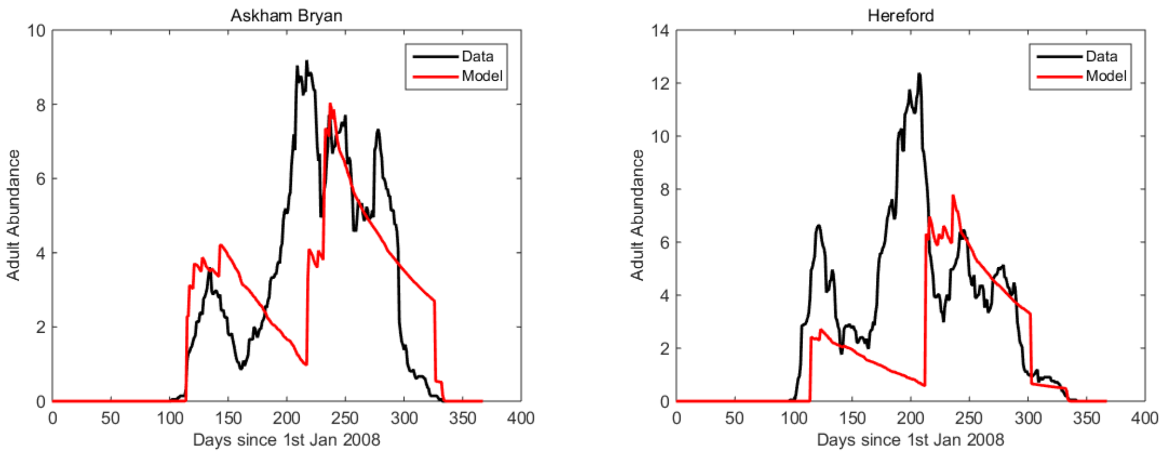

[2] http://uk.mathworks.com/matlabcentral/fileexchange/24298-minimize

[3] © 2015 The MathWorks, Inc. MATLAB and Simulink are registered trademarks of The MathWorks, Inc.

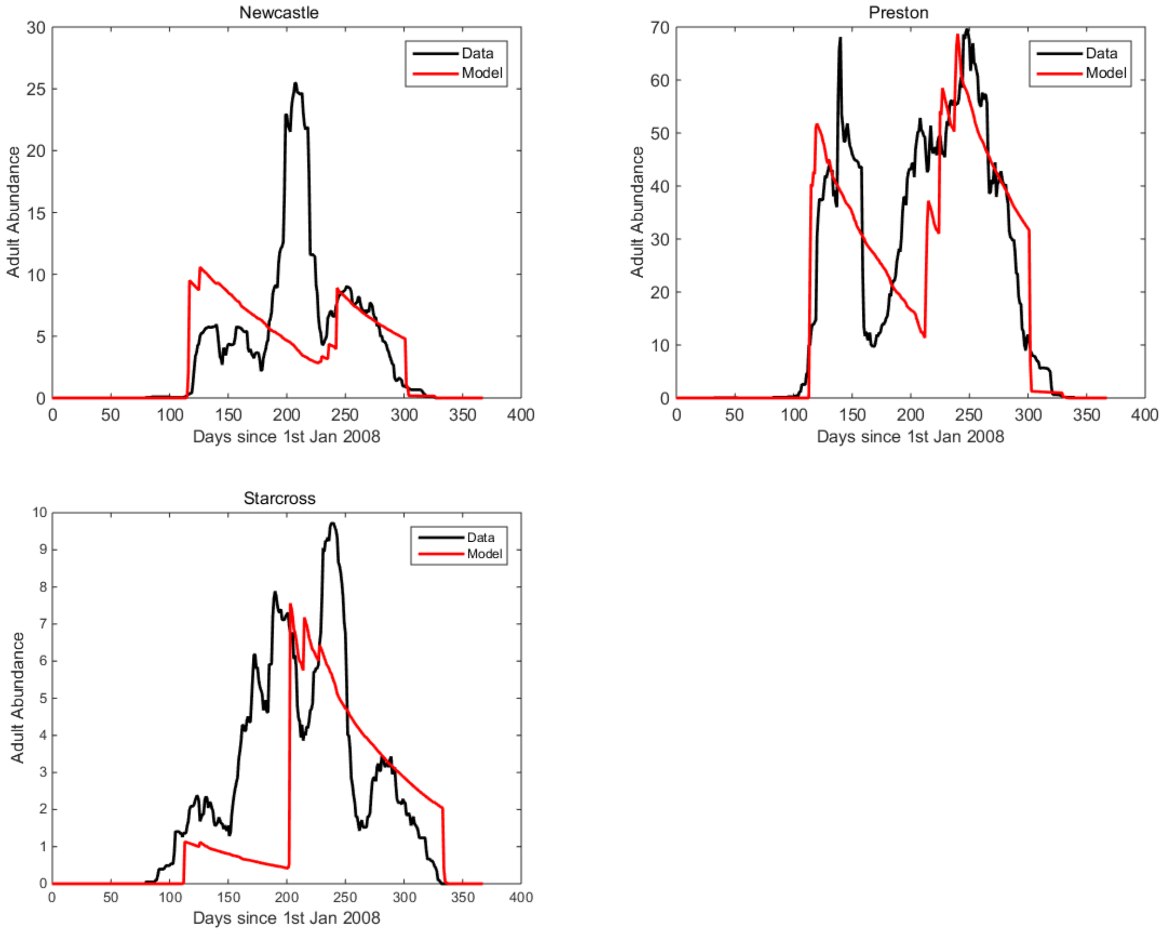

Figure S2: Plots showing the results of the least squares fitting across all five sites. The data (in black), the total number of Obsoletus group adults, has been interpolated to account for missing values and smoothed using a 1-week moving window. The model output (in red) has also been smoothed using a 1-week moving window.

Table S1: Results of the deterministic model least squares fitting. The density-dependence parameters are site specific, whereas the diapause and scaling factor parameters apply to all sites.

	1 Askham Bryan	2 Hereford	3 Newcastle	4 Preston	5 Starcross	Site unspecific
**Density-dependence coefficient, a_i**	2.21E-09	1.98E-07	1.24E-07	1.33E-08	3.19E-07	-
**Density-dependence coefficient, b**	0.7289	3.3399	4.5299	2.6759	5.6964	-
**Scaling factor**	-	-	-	-	-	8.64E-05
**Diapause at start of year, d_{start}**	-	-	-	-	-	46986.50
**Diapause at end of year, d_{end}**	-	-	-	-	-	49065.52

ABC Fitting

So that the full stochastic model can be fitted to the data, we implement an approximate Bayesian computational method (ABC). This method is a statistically rigorous technique for estimating how well different models and parameterisations are supported by the available data, given some prior beliefs about how likely they are. ABC involves running models a large number of times, with parameters drawn randomly from their prior distributions, and then retaining the simulations closest to the observations [1, 2, 3, 4].

For the prior distributions of each parameter, we choose uniform distributions, centred on the values found previously from the least squares fitting, except the diapause parameters which have a uniform distribution over the permitted values (see Figure S3). The stochastic model is run for 600000 runs with parameters selected at random from the prior distributions.

We used an R^2 distance measure as this gave the best model fit. An absolute and square root of the summed squared distances measures were considered but gave a lesser model fits. The R^2 distance measure $\rho(m_i, D)$ is given by

$$\rho(m_i, D) = 1 - \frac{\sum_j (m_{i,j} - D_j)^2}{\sum_j (D_j - \bar{D})^2}.$$

In this equation, i is the model run, D_i is the data point, mi, j is run i's output for data point j, Dj is the empirical data for data point j, and $\bar{D}$ is the mean of the empirical data.

Table S2: Results of the stochastic ABC fitting. We give the statistics on the spread of the posterior parameters. Site 1=Askham Bryan, 2=Hereford, 3=Newcastle, 4=Preston and 5=Starcross.

	a_1	b_1	a_2	b_2	a_3	b_3	a_4	b_4	a_5	b_5	c	d_{start}	d_{end}
**Min**	8.84E-10	0.30	8.45E-08	1.65	5.01E-08	1.37	5.66E-09	1.39	1.57E-07	2.44	4.35E-05	4.36E+04	4.50E+04
**2.5%**	1.01E-09	0.32	1.20E-07	2.04	5.98E-08	1.97	1.05E-08	1.81	2.15E-07	3.90	6.60E-05	4.40E+04	4.52E+04
**Median**	2.76E-09	0.64	2.84E-07	4.47	2.10E-07	5.30	2.96E-08	3.83	4.27E-07	11.09	1.25E-04	4.55E+04	4.85E+04
**Mean**	2.77E-09	0.62	2.79E-07	4.48	2.08E-07	5.41	2.92E-08	3.77	4.27E-07	10.82	1.26E-04	4.56E+04	4.85E+04
**97.5%**	4.33E-09	0.84	3.92E-07	6.56	3.40E-07	8.78	4.59E-08	5.26	6.19E-07	16.58	1.92E-04	4.81E+04	5.29E+04
**Max**	4.39E-09	1.10	3.97E-07	6.68	3.47E-07	9.04	4.65E-08	5.35	6.36E-07	17.09	2.15E-04	4.93E+04	5.36E+04

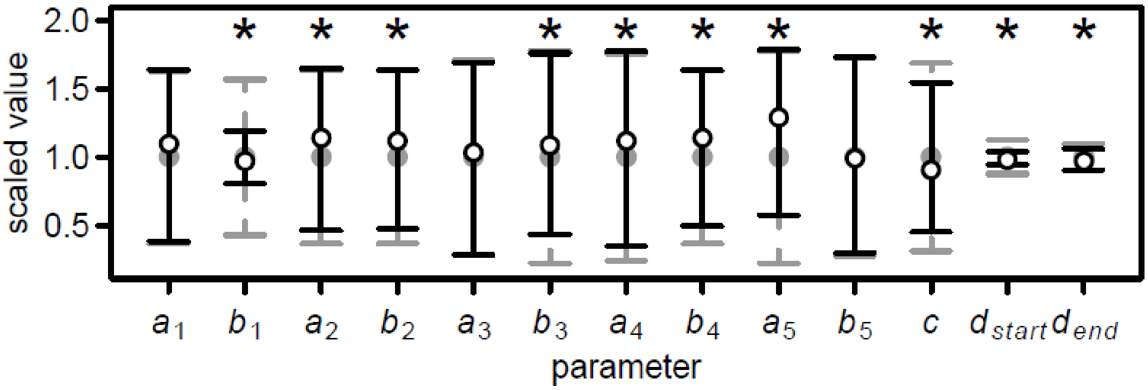

Figure S3: Prior and posterior distributions from the ABC fitting. Points show the median value; posterior distributions are shown in black; priors are shown by grey dashed lines. Asterisks mark significant narrowing ($p < 0.01$) after correcting for multiple testing. All parameter values were scaled by dividing by the median of the corresponding prior, thus identifying relative narrowing in the median. The density-dependence parameter indices are ordered in ascended alphabetical order with site 1=Askham Bryan, 2=Hereford, 3=Newcastle, 4=Preston and 5=Starcross.

ABC has been used to select the 1000 ‘best’ runs out of 100000 runs of the midge model. These accepted runs are those with the smallest difference to the data. Of these 1000 best-fitting samples, the posterior distributions of the 13 input parameters are shown in Figure S3. This figure shows that the majority of parameters have narrowed, which indicates that the posterior distribution more accurately describes the data.

Out of the 1000 accepted runs, Figure S4 shows the frequency distribution of parameter values occurring in the accepted runs. The plots reveal that some distributions are skewed, whilst other exhibit a more centralised and narrowed distribution.

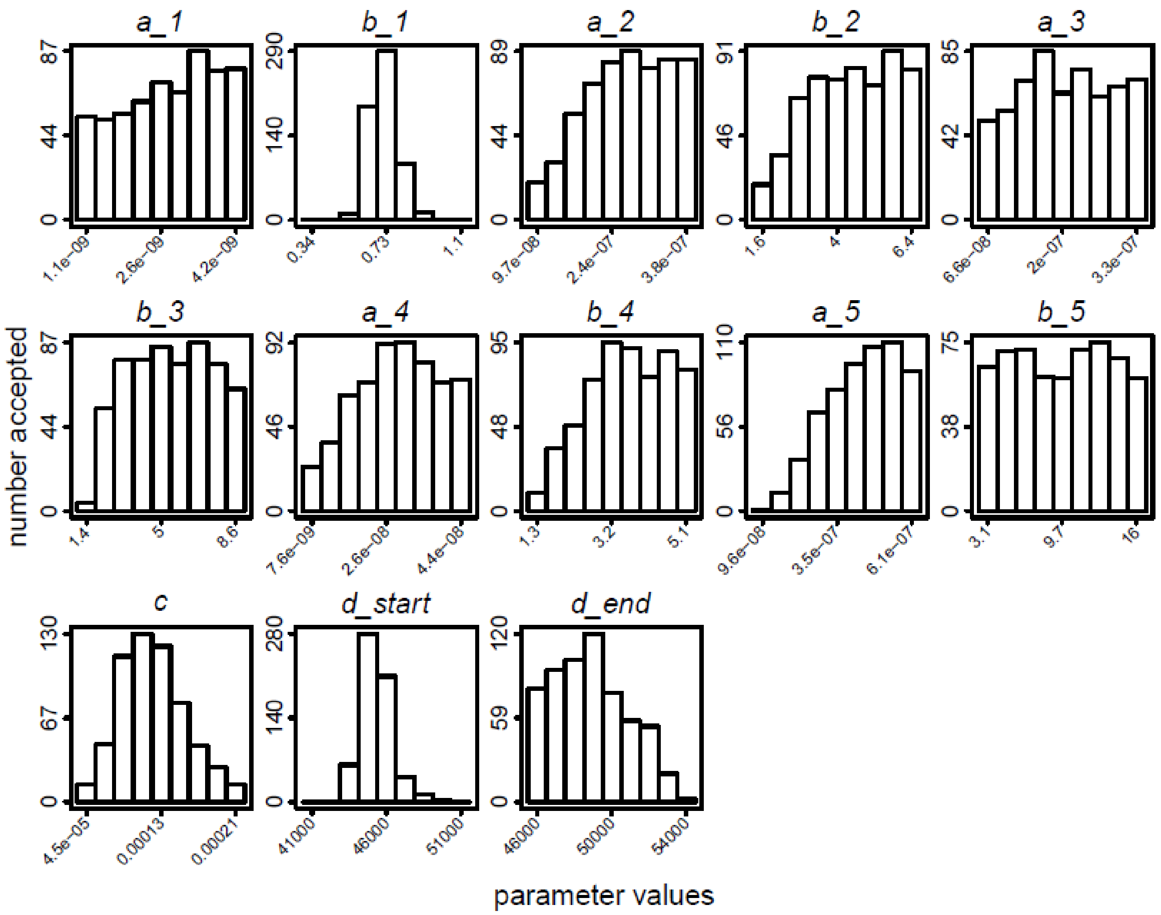

Figure S4: Posterior parameter distributions. The 1000 accepted runs are binned into 10 column bins, thus giving the posterior parameter distribution. The density-dependence parameter indices are ordered in ascended alphabetical order with site 1=Askham Bryan, 2=Hereford, 3=Newcastle, 4=Preston and 5=Starcross.

Finally, we compare the fitted model to the data (see Figure S5). Here we have plotted 100 (randomly chosen) of the accepted runs, the best run and the data for each of the sites. It shows that the model fits the data reasonably well (see main text for further discussion).

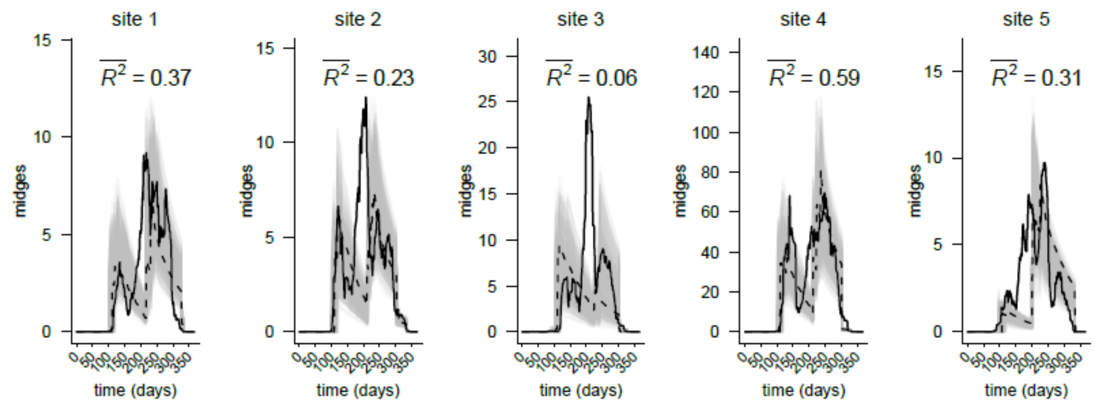

Figure S5: Comparison between fitted model runs and the data. Here we have plotted in grey 100 of the accepted runs alongside the data (black line) and the single best run (black dashed line) for each of the sites. The density-dependence parameter indices are ordered in ascended alphabetical order with site 1=Askham Bryan, 2=Hereford, 3=Newcastle, 4=Preston and 5=Starcross.

We conclude that the ABC model fitting gives a fit and we therefore use the mean parameter values for further model scenario testing and modelling control strategies (see main text).

References

1. Beaumont MA. Approximate Bayesian Computation in Evolution and Ecology. Annu Rev Ecol Evol Syst. 2010;41 1:379-406.
2. Csillery K, Blum MGB, Gaggiotti OE, Francois O. Approximate Bayesian Computation (ABC) in practice. Trends Ecol Evol. 2010;25 7:410-8.
3. Toni T, Welch D, Strelkowa N, Ipsen A, Stumpf MPH. Approximate Bayesian computation scheme for parameter inference and model selection in dynamical systems. J R Soc Interface. 2009;6 31:187-202.
4. Hartig F, Calabrese JM, Reineking B, Wiegand T, Huth A. Statistical inference for stochastic simulation models – theory and application. Ecol Lett. 2011;14 8:816-27.
5. Sanders CJ, Shortall CR, Gubbins S, Burgin L, Gloster J, Harrington R, et al. Influence of season and meteorological parameters on flight activity of *Culicoides* biting midges. J Appl Ecol. 2011;48 6:1355-64.
6. Hartemink NA, Purse BV, Meiswinkel R, Brown HE, de Koeijer A, Elbers AR, et al. Mapping the basic reproduction number (R(0)) for vector-borne diseases: a case study on bluetongue virus. Epidemics. 2009;1 3:153-61.
